# High-risk human papillomavirus detection in self-collected vaginal samples compared with healthcare worker collected cervical samples among women attending gynecology clinics at a tertiary hospital in Pretoria, South Africa

**DOI:** 10.1186/s12985-021-01662-5

**Published:** 2021-09-23

**Authors:** Teboho Amelia Tiiti, Tebogo Loraine Mashishi, Varsetile Varster Nkwinika, Ina Benoy, Selokela Gloria Selabe, Johannes Bogers, Ramokone Lisbeth Lebelo

**Affiliations:** 1 Department of Virological Pathology, Sefako Makgatho Health Sciences University, Pretoria, South Africa; 2 Laboratory of Cell Biology and Histology, Faculty of Medicine and Health Sciences, University of Antwerp, Antwerpen, Belgium; 3 South African Vaccination and Immunization Centre, Sefako Makgatho Health Sciences University, Pretoria, South Africa; 4 Algemeen Medisch Laboratorium (AML), Sonic Healthcare, Antwerpen, Belgium; 5 National Health Laboratory Service/Department of Virological Pathology, Sefako Makgatho Health Sciences University, Pretoria, South Africa; 6 Department of Anatomical Pathology, Sefako Makgatho Health Sciences University, Pretoria, South Africa

**Keywords:** Cervical cancer, Human papillomavirus, HPV-self-sampling, ThinPrep PreservCyt, HPV mRNA testing

## Abstract

**Background:**

In 2017, the South African National Department of Health (NDoH) Cervical Cancer Prevention and Control Policy was revised. Human papillomavirus (HPV) testing on self-collected samples may offer improved screening uptake. The objectives of the study were to compare the positivity of high-risk (hr)-HPV deoxyribonucleic acid (DNA) and hrHPV viral messenger ribonucleic acid (mRNA) between healthcare worker-collected cervical and self-collected vaginal samples and investigate the accuracy of the applicator-tampon-based self-collected samples in detecting hrHPV DNA and hrHPV mRNA.

**Methods:**

A total of 527 women aged 18 years and older and seeking gynecology services at a tertiary hospital in Pretoria, South Africa, were enrolled. Vaginal samples were self-collected using SelfCerv applicator tampon, followed by cervical samples collected by a healthcare worker using a Cervex Brush® Combi. Both samples were tested with the Abbott m2000 analyzer for 14-hrHPV types and 285 paired samples were tested for hrHPV E6/E7 mRNA using the Aptima HR-HPV mRNA assay. The prevalence of hrHPV DNA and hrHPV E6/E7 mRNA was estimated and the positivity between the two collection methods was compared for the total group as well as per age group.

**Results:**

HrHPV prevalence was 48.0% (95% CI 43.7–52.4) among healthcare worker collected samples and 47.6% (95% CI 43.3–52.0) among self-collected samples. There was no difference in positivity between healthcare worker collection (48.0%) and applicator-tampon-based self-collection, 47.6% (p-value = 0.90). The proportions of hrHPV were equal between the age groups as shown by the McNemar test (p = 0.9036) results for correlated proportions. The prevalence of hrHPV mRNA was 78.6% (95% CI 73.4–83.2) and 58.6% (95% CI 52.6–64.4) for healthcare worker- and self-collection, respectively. The McNemar test for correlated proportions was highly significant (p < 0.0001), indicating that the hrHPV mRNA proportions are not comparable, although this differed between age groups.

**Conclusions:**

Applicator-tampon-based self-collection has a comparable hrHPV DNA positivity rate as healthcare worker collection but different positivity rates for hrHPV mRNA. Self-sampling showed high concordance with healthcare worker-collected sampling for hrHPV DNA detection, especially regarding HPV 16/18 detection. HrHPV DNA was equally detected between the total group as well as per age group. Implementation of self-sampling using an applicator tampon as a primary screening tool may be considered.

## Background

Cervical cancer is the third most common cancer in women worldwide, with an estimated 570,000 new cases and 311,000 deaths reported in 2018 [1]. The incidence and mortality of cervical cancer have reduced in high-income countries (HICs) following the implementation of regular screening programs and annually repeated Papanicolaou (Pap) smear analysis [2]. However, cervical cancer remains a health problem in low- and middle-income countries (LMICs) as cytology-based screening programs have not been effective in reducing the cervical cancer burden for different reasons such as low screening coverage, lack of quality assurance, and poor organization of government screening programs [2]. Cervical cytology is very specific for the detection of precancerous lesions or cancer [3]. However, false-negative results are consequently caused by sampling and detection errors and the sensitivity as low as 30% [4]. According to the National Department of Health (NDoH) Cervical Cancer Prevention and Control Policy of South Africa, factors contributing to high cervical cancer incidence in the country include socioeconomic status and place of residence, education level, and social arrangement of the family, access to services, healthcare worker skills, and stigma [5].

The South African National Department of Health has had a cervical cancer screening program since 2000 and makes provision for three cervical cancer screening tests in 10-year intervals for HIV-negative asymptomatic women aged 30 years and older. The policy has since been updated to include primary prevention of cervical cancer through HPV vaccination of young girls aged 9–12 years [5]. Furthermore, the policy has taken into consideration the availability of new screening technologies and plans to institutionalize quality assurance for screening approaches to foster the accuracy and reliability of the tests. In terms of new screening technologies available, the government has proposed the rollout of HPV testing and recommends the investigation of self-sampling for HPV testing [5].

HPV testing allows for the opportunity of self-collected samples. Moreover, several studies have demonstrated comparable HPV results between self-collected and healthcare worker-collected samples [6–8]. Comparable HPV results between self-collected samples and healthcare worker-collected samples have resulted in the use of HPV testing on self-collected samples as a primary screening modality in HICs. Furthermore, findings from a meta-analysis suggest that self-sampling for HPV may increase screening uptake [9]. Additionally, several studies that have offered HPV self-sampling have shown an increase in participation rates [10–13]. Participation rates may be increased because of not undergoing a pelvic examination, which is one common barrier to screening [14]. Factors that may discourage women from attending regular screening include lack of time, discomfort, inconvenience, and cultural objections [15]. Therefore, self-sampling may cater to the mentioned factors and thus, it may improve screening coverage.

To date, there is inadequate evidence on the comparative performance of self-sampling devices in South Africa. In the revised South African Cervical Cancer Prevention and Control Policy mentioned above, data is required to support the inclusion of self-sampling in the context of national cervical cancer screening programs in the country. The SelfCerv applicator tampon (Ilex Medical Ltd, Johannesburg, South Africa) is an improved, convenient, discreet, and easy-to-use device that is intended to self-collect vaginal samples for the identification of HPV and thus, remains to be investigated in terms of HPV detection. The objectives of the study were to compare the positivity of hrHPV DNA and hrHPV mRNA between healthcare worker-collected cervical and self-collected vaginal samples and investigate the accuracy of the applicator-tampon-based self-collected samples in detecting hrHPV DNA and hrHPV mRNA.

## Methods

### Study design and population

A cross-sectional study was conducted from June 2016 to November 2018 among 527 women attending gynecology clinics at a tertiary hospital in Pretoria, South Africa. The patients attended the gynecology clinic for review with pap test after treatment with large loop excision of the transformation zone (LLETZ); colposcopy examination after a positive pap test, cervical cancer screening using a pap test; and termination of pregnancy, family planning, and other gynecology-related services.

### Inclusion and exclusion criteria

The study included women who were 18 years and older. Women who had undergone a complete hysterectomy or were going through their menstrual cycle at the time of the study were excluded.

### Specimen collection

The study researcher explained the study and instructed participants on how to perform the applicator-tampon-based self-collection. Participants were handed a questionnaire to collect socio-demographic and clinical characteristics. Thereafter, participants were then instructed to fill in the questionnaire and leave out the questions on self-collection and only fill after both procedures were performed. The SelfCerv, a medium-sized applicator tampon, was used for self-collection. Briefly, consenting participants were individually taken to a private room and asked to collect a vaginal sample by inserting the applicator tampon into the vagina for two hours and return to the waiting room. After two hours, the participants were requested to remove the tampon and place the tampon in the provided specimen bottle solution that contained saline buffer, taking care not to touch the tampon with their hands.

Following self-collection, a healthcare worker then performed a pelvic examination. A sterile speculum was inserted to visualize the cervix and a broom-like collection device, Cervex Brush® Combi (Rovers Medical Devices, B.V., The Netherlands) was inserted and rotated two full turns clockwise. The brush containing endocervical cells was then rinsed in the ThinPrep PreservCyt Solution (Hologic Inc., Marlborough, United States of America) to dislodge cervical cells and discarded. Both samples were transported at room temperature to the HPV and STIs Training Centre for Africa at Sefako Makgatho Health Sciences University for analysis. A volume of 20 mL of ThinPrep PreservCyt solution was added to the specimen bottle containing the applicator tampon upon arrival. The bottle is made such that it is easy to squeeze to remove the tampon, as the tampon string was already on the outside of the bottle. The bottle was swirled; the tampon was squeezed, removed, and discarded. The solution was thereafter, transferred into an empty ThinPrep vial and labelled.

### Abbott RealTime High-Risk HPV testing

The Abbott RealTime High-Risk HPV assay (Abbott Molecular GmbH & Co. KG, Wiesbaden, Germany) is a real-time PCR assay that detects 14 hrHPV DNA (16, 18, 31, 33, 35, 39, 45, 51, 52, 56, 58, 59, 66 and 68) and genotypes only HPV types 16 and 18 from the other 12 hrHPV (non-HPV 16/18) types. A volume of 800 µL of healthcare worker and self-collected samples was transferred into Abbott m2000 Master-Mix tubes, respectively, and loaded into the Abbott m2000sp (Sample Preparation) system for extraction using the mSample Preparation System DNA kit. Samples were then amplified and hrHPV genotyped using the Abbott RealTime hrHPV on the Abbott m2000rt (RealTime) according to the manufacturer's instructions. The endogenous human beta-globin sequence was detected as sample validity control for cell adequacy. Results were reported as either positive or negative. Positive results were further described as HPV-16, HPV-18, or ‘Other hrHPV’.

### High-risk HPV mRNA testing

A total of 285 healthcare worker and/or self-collected paired samples tested positive for hrHPV on either sample (218 hrHPV self-collected positive and healthcare worker-collected positive, 32 hrHPV self-collected positive and healthcare worker-collected negative, and 35 hrHPV self-collected negative and healthcare worker positive) were tested for HPV mRNA. An aliquot of 1 mL was transferred from the ThinPrep vial container to an Aptima Specimen Transfer tube containing the medium and tested using APTIMA® HPV assay *(*Hologic Gen-Probe, Inc., San Diego, Canada) according to the manufacturer's instructions. The APTIMA® HPV assay is a target amplification nucleic acid probe test for the in vitro qualitative detection of E6/E7 mRNA from 14 hrHPV types (16, 18, 31, 33, 35, 39, 45, 51, 52, 56, 58, 59, 66 and 68). The assay did not discriminate between the 14 high-risk types, and the assay results were interpreted based on the signal/cutoff ratio for the analyte.

### Statistical analysis

Prevalence estimates are presented with 95% confidence intervals. The overall hrHPV DNA and hrHPV mRNA detection rates between the two collection methods were compared. A p-value of 0.05 and less was considered statistically significant. Concordance was calculated, and a McNemar test for correlated proportions comparing the positive hrHPV and/or hrHPV mRNA of self-collected samples with the positive hrHPV and/or hrHPV mRNA of healthcare worker-collected samples for the total group as well as per age group was performed. All statistical analyses were performed using STATA version 14.1 (StataCorp, College Station, Texas).

## Results

### Participant characteristics

The mean (± standard deviation [SD]) age for enrolled women was 36.8 (± 11.0) years. The age of the participants ranged from 18 to 68 years. A greater proportion (32.6%) of participants were in the age group between 30 and 39 years, followed by those aged less than 30 years (28.7%). Two-thirds (67.2%) of the participants were single and more than half (54.8%) were unemployed. The majority (85.0%) of the participants were from semi-urban areas (Table 1).

**Table 1:** Demographic characteristics of the study participants

	n	%
Age in years		
18–29	151	28.7
30–39	172	32.6
40–49	121	23.0
50–59	61	11.6
≥ 60	19	3.6
Unspecified	3	0.6
Marital status		
Single	354	67.2
Married	126	23.9
Divorced/widowed/separated	46	8.7
Unspecified	1	0.2
Employment status		
Employed	237	45.0
Unemployed	289	54.8
Unspecified	1	0.2
Place of residence		
Rural	12	2.3
Semi-urban	448	85.0
Urban	63	12.0
Unspecified	4	0.8

### HPV DNA detection

Amplifiable DNA extraction was successfully achieved on all healthcare worker-collected and self-collected samples as shown with a positive internal control. There was no statistical significant difference in the rate of positivity for hrHPV DNA between the healthcare worker-collected samples [48.0% (95% CI 43.7–52.4)] and self-collected samples [47.6%, 95% CI 43.3–52.0)], p-value = 0.90. The overall concordance between self-collected samples and healthcare worker-collected samples was good (87.1%). The proportion of true positive self-collected samples was 86.2% (218/253; 95%CI, 81.3–90.2), while the proportion of true negative self-collected samples was 88.0% (241/274; 95% CI, 83.5–91.6) using the healthcare worker-collected samples as reference. The McNemar test for correlated proportions was not significant (p = 0.9036), indicating that the proportions are comparable (Table 2).

**Table 2:** Prevalence of hrHPV between self and healthcare worker-collected samples

		Self-sampling		Total (%)
		hrHPV positive (%)	hrHPV negative (%)	
Healthcare worker-sampling	hrHPV positive (%)	218 (41.4)	35 (6.6)	253 (48.0)
	hrHPV negative (%)	33 (6.3)	241 (45.7)	274 (52.0)
	Total (%)	251 (47.6)	276 (52.4)	527 (100.0)

McNemar test for correlated proportions comparing hrHPV positive portion of self-sampling with a positive portion of healthcare worker sampling, p = 0.9036

The hrHPV DNA detection rate in both healthcare worker-collected and self-collected samples decreased with increasing age from 57.0% in the age group ≤ 29 years, to 36.8% in the age group ≥ 60 years (Table 3). The McNemar test for correlated proportions was not significant, indicating that the proportions are comparable between the age groups.

**Table 3:** Detection of hrHPV categorized by age group

Age category (in years)	Participants per age group	Healthcare worker-sampling		Self-sampling		P-value*
		hrHPV Positive (%)	hrHPV Negative (%)	hrHPV Positive (%)	hrHPV Negative (%)	
≤ 29	151	86 (57.0)	65 (43.0)	86 (57.0)	65 (43.0)	1.0000
30–39	172	78 (45.3)	94 (54.7)	76 (44.2)	96 (55.8)	0.8145
40–49	121	59 (48.8)	62 (51.2)	59 (48.8)	62 (51.2)	1.0000
50–59	61	23 (37.7)	38 (62.3)	23 (37.7)	38 (62.3)	1.0000
≥ 60	19	7 (36.8)	12 (63.2)	7 (36.8)	12 (63.2)	1.0000
Unknown	3	–	3(100.0)	–	3 (100.0)	1.0000
Total	527	253 (48.0)	274 (52.0)	251 (47.6)	276 (52.4)	0.9036

*McNemar test for correlated proportions

### HPV genotyping

Table 4 shows the results for HPV detection separating HPV16/18 from the ‘Other hrHPV’ types (non-HPV16/18). The concordance for HPV16/18 positives was 93.9%, with 16.5% of HPV 16/18 positives in self-sampling and 15.4% in healthcare worker sampling groups. For non-HPV 16/18, the concordance was 86.5%, with no difference between non-HPV16/18 positives in self-collected (42.1%) and healthcare worker-collected samples (41.9%). The McNemar test for correlated proportions was not significant for both HPV16 and/or 18 (p = 0.38) and non-HPV 16/18 positives (p = 1.0000), indicating that the proportions were comparable. Of the total healthcare worker-collected samples, 62 (11.8%) showed single or multiple infections with genotype HPV-16; 19 samples (3.6%) showed single or multiple infections with genotype HPV-18 excluding any coinfections with genotype HPV-16. In self-collected samples, 12.0% showed single or multiple infections with genotype HPV-16 and 4.6% of samples were positive with single or multiple infections with genotype HPV-18 (excluding coinfections with HPV-16). Thirty-six out of 71 discordant samples were positive for non-16/18 HPV genotype in self-collected samples and negative in healthcare worker-collected samples, and 35 samples were negative for non-16/18 HPV genotype in self-collected samples and positive in healthcare worker-collected samples.

**Table 4:** Prevalence of HPV 16/18 and non-16/18 hrHPV

		Self-sampling		Total (%)
		HPV 16/18 positive	HPV 16/18 negative	
Healthcare worker-sampling	HPV 16/18 positive (%)	68 (12.9)	13 (2.5)	81 (15.4)
	HPV 16/18 negative (%)	19 (3.6)	427 (81.0)	446 (84.6)
	Total (%)	87 (16.5)	440 (83.5)	527 (100.0)

		Non-16/18 hrHPV positive	Non-16/18 hrHPV negative	
Healthcare worker-sampling	Non-16/18 hrHPV positive (%)	186 (35.3)	35 (6.6)	221 (41.9)
	Non-16/18 hrHPV negative (%)	36 (6.8)	270 (51.2)	306 (58.1)
	Total (%)	222 (42.1)	305 (57.9)	527 (100.0)

McNemar test for correlated proportions, HPV 16/18 positive samples, p = 0.38

McNemar test for correlated proportions non-16/18 hrHPV positive samples, p = 1.0000

### HPV mRNA detection

Table 5 illustrates the hrHPV mRNA prevalence of healthcare worker-collected and self-collected samples. There was a significant difference in the rate of positivity for hrHPV mRNA between healthcare worker- (78.6%, 95% CI 73.4–83.2) and self-collected samples (58.6%, 95% CI 52.6–64.4), p < 0.0001). The overall concordance between self-collected samples and healthcare worker-collected samples was 70.2%.

**Table 5:** Prevalence of hrHPV mRNA between self-and healthcare worker-collected samples

		Self-sampling		Total (%)
		hrHPV mRNA positive (%)	hrHPV mRNA negative (%)	
Healthcare worker-sampling	hrHPV mRNA positive (%)	153 (53.7)	71 (24.9)	224 (78.6)
	hrHPV mRNA negative (%)	14 (4.9)	47 (16.5)	61 (21.4)
	Total (%)	167 (58.6)	118 (41.4)	285 (100.0)

McNemar test for correlated proportions comparing hrHPV positive portion of self-sampling with a positive portion of healthcare worker sampling, p =  < 0.0001

The McNemar test for correlated proportions was highly significant (p < 0.0001), indicating that the proportions of hrHPV mRNA are not comparable, although this differed between age groups (Table 6). HrHPV mRNA increased with increasing age in women aged ≤ 29 to 49 years. Significant differences were observed in women between the ages of 30–49 years.

**Table 6:** Detection of hrHPV mRNA categorized by age group

Age category (in years)	Participants per age group	Healthcare worker-sampling		Self-sampling		P-value*
		hrHPV mRNA Positive (%)	hrHPV mRNA Negative (%)	hrHPV mRNA Positive (%)	hrHPV mRNA Negative (%)	
≤ 29	99	64 (64.6)	35 (35.4)	53 (53.5)	46 (46.5)	0.0614
30–39	84	70 (83.3)	14 (16.7)	52 (61.9)	32 (38.1)	0.0001
40–49	67	61 (91.0)	6 (9.0)	41 (61.2)	26 (38.8)	< 0.0001
50–59	28	23 (82.1)	5 (17.9)	16 (57.1)	12 (42.9)	0.0654
≥ 60	7	6 (85.7)	1 (14.3)	5 (71.4)	2 (28.6)	1.0000
Total	285	224 (78.6)	61 (21.4)	167 (58.6)	118 (41.4)	< 0.0001

*McNemar test for correlated proportions

## Discussion

HICs have been able to reduce cervical cancer incidence and mortality; however, LMICs continue to bear the burden of the disease [16]. Furthermore, in HICs, HPV as the primary test for cervical cancer screening has gradually been introduced [17]. Although HPV testing can be done using a self-collected specimen (self-sampling), which can potentially improve the uptake of screening in LMICs, challenges such as autonomy, cost, and limited health care resources need to be addressed [18]. Regardless of the challenges of introducing HPV testing, it is important to evaluate the opportunities for introducing HPV testing as the primary screening method in LMICs. In Sub-Saharan Africa, particularly South Africa, the majority of studies have investigated women's perceptions, acceptability, and preference of HPV self-sampling [19–23] in comparison to healthcare worker sampling. However, studies comparing the detection rate of HPV between healthcare worker-collected- and self-collected samples in LMICs are limited. Moreover, the majority of studies have focused on comparing healthcare worker-collected and self-collected samples for HPV DNA detection [7, 8, 24–26] than HPV mRNA detection.

This study aimed to compare the positivity of hrHPV DNA and hrHPV mRNA between healthcare worker-collected and self-collected samples and investigate the accuracy of the applicator-tampon-based self-collected samples in detecting hrHPV DNA and hrHPV mRNA. Although healthcare worker-collected samples detected two more hrHPV DNA positives than self-collected samples, the study found no statistical difference in the hrHPV DNA positivity rate between the two collection methods. Comparable to previous studies, similar findings have been reported [27, 28]. However, several studies have reported a slightly high HPV detection rate in self-collected samples than healthcare worker-collected samples [7, 25, 29, 30]. The differences in the detection rates between both samples may be attributed to the different self-sampling devices and HPV testing methods used. Studies that have used brush self-samplers (Evalyn brush, Dacron swab, and cytobrush), which mainly collect cervical and vaginal cells, have shown a higher detection rate. In addition, some of the studies that have reported a higher detection rate for HPV DNA have used testing platforms that detect more than 14 HPV types compared to the platform used in the current study. Furthermore, the McNemar test for correlated proportions did not indicate a difference in hrHPV positivity among the age categories. Although there are limited studies that have reported the accuracy of hrHPV detection stratified by age between healthcare worker-collected and self-collected samples, Ketelaars et al. indicated differing proportions between age groups using an Evalyn brush with the Roche Cobas 4800 hrHPV test [30].

The most oncogenic hrHPV genotypes are HPV-16 and HPV-18, reported in more than 70% of all cervical cancer cases [31]. Therefore, the detection of HPV-16 and/or HPV-18 identifies women at the greatest risk of high-grade lesions [32]. The Abbott m2000 system makes a distinction between non-16/18 HPV (31, 33, 35, 39, 45, 51, 52, 56, 58, 59, 66, and 68) and HPV-16 and HPV-18. In this study, HPV-16 and HPV-18 were equally detected in healthcare worker-collected and self-collected samples. Non-HPV-16/18 (Other hrHPV genotypes) were equally detected in both methods with the self-collection detecting one more sample compared to the health worker collection. Previous studies have reported similar findings [7, 8]. The discordance between healthcare worker-collected and self-collected samples was not notable for non-HPV-16/HPV-18. In contrast, Ketelaars et al. reported a notable discordance for non-HPV-16/18 positive samples in the self-sampling group versus negative samples in the healthcare worker-collected group [30].

As indicated in the methods and results, not all 527 were tested for hrHPV mRNA, only samples that were positive in either healthcare worker-collected and/or self-collected samples were tested. It is acknowledged that this may have introduced bias in terms of samples selected, which may have influenced the results. However, the number of hrHPV DNA discordant samples was not different between healthcare worker-collected and self-collected samples. Therefore, the significant difference observed in hrHPV mRNA positivity rate between healthcare worker-collected and self-collected samples might be a true reflection of the results, which shows that healthcare worker-collected samples have an increased detection rate for hrHPV mRNA. Similar to previous studies, healthcare worker-collected samples have a high detection rate compared to self-collected samples [33, 34]. Our findings are different from those reported by Adamson et al. who found no difference in the rate of positivity between the two collection methods [35]. It is uncertain if the positivity rate differences reported by Adamson et al. and in our study may be due to the technicality of the tampons, an applicator tampon was used in the current study compared to a non-applicator tampon used in Adamson et al. study. The order of sample collection was also different as self-collection was performed first in the current study compared to Adamson et al. who collected healthcare worker samples first [35]. The discrepancy may also be explained by incorrectly collected self-samples, incorrect transfer or storage of the samples, and an inadequate amount of cells, which may lead to the lower sensitivity of vaginal HPV self-sampling. South African studies have used the non-applicator tampon device [22, 29, 35, 36] hence, women in the general population are more familiar with the non-applicator tampons compared to the applicator tampon used in this study.

Seventy-one (24.9%) of the samples which tested negative for hrHPV in self-collected samples, tested positive in the healthcare worker-collected samples. The lower positivity rate observed in self-collected samples might be due to HPV mRNA quantities being below the analytical sensitivity of the Aptima assay due to the insufficient amount of cells, as the applicator tampon might have not collected sufficient cells from the transformation zone. A true difference was observed in women aged between 30–39 and 40–49 years. Considering a significant difference in the detection of hrHPV mRNA in self-collected samples compared to healthcare worker-collected samples and consequently, hrHPV mRNA possibly being a more accurate and specific screening tool to detect women at higher risk of cervical cancer development, it would be interesting to investigate measures to increase the positivity rate in self-collected samples. Hence, a different protocol on previously negative hrHPV mRNA self-collected samples has been trialled by Borgfeldt and Forslund [37]. An additional step (90℃ for 1 h) on specimens stored in the Aptima media has been investigated and shown to return a positive result to a previously negative sample [37]. Therefore, HPV mRNA-based studies are needed to investigate if the mRNA amplification process in self-collected vaginal samples is associated with lower detection rates. Caution in the interpretation of the findings of the study concerning the study's limitations would include the fact that 242 samples were excluded for hrHPV mRNA testing. Our population being a high-risk population, the results may not be generalizable to the whole population.

## Conclusions

Self-sampling with the SelfCerv applicator tampon compared with the Abbott m2000 analyzer showed a high concordance and comparable hrHPV detection as in healthcare worker-collected samples, especially regarding HPV 16/18 detection. More non-16/18 HPV genotype infections were detected with self-sampling than healthcare worker sampling. Self-collected samples demonstrated a lower positivity rate for the detection of hrHPV mRNA. HPV DNA self-sampling using the SelfCerv applicator tampon in women attending gynecology clinics as a primary screening tool may be considered.

## Data Availability

The datasets used and analyzed during the current study are available from the corresponding author on reasonable request.

## Abbreviations

HPV: Human papillomavirus
Hr-HPV: High-risk Human papillomavirus
LLETZ: Large loop excision of the transformation zone
CI: Confidence interval
